# Reviewer acknowledgement 2014

**DOI:** 10.1186/s12941-015-0068-2

**Published:** 2015-04-19

**Authors:** Hakan Leblebicioglu

**Affiliations:** Ondokuz Mayis University Medical School, Samsun, Turkey

## Abstract

*Annals of Clinical Microbiology and Antimicrobials* would like to thank the following colleagues for their assistance with peer review of manuscripts for the journal in 2014.

**Oladipo Aboderin**

Nigeria

**Ayaz Ahmed**

Pakistan

**Halis Akalin**

Turkey

**Ramazan Akdemir**

Turkey

**Mohd Sayeed Akhtar**

Malaysia

**Mohamed Al-Agamy**

Saudi Arabia

**Werner C. Albrich**

Switzerland

**Nafisa Ali**

Pakistan

**Franz Allerberger**

Austria

**Ayman Al-Mariri**

Syria

**Khalid Almas**

United States of America

**Muna Almaslamani**

Qatar

**Mustafa Altindis**

Turkey

**Paula Anibal**

Brazil

**Fabiana Antognoni**

Ireland

**Paulrayer Antonisamy**

South Korea

**Asghar Arshi**

Iran

**Ferhat Arslan**

Turkey

**Marise Asensi**

Burkina Faso

**Vildan Avkan Oguz**

Turkey

**Babafela Awosile**

Canada

**Hande Aydemir**

Turkey

**Andrea Babic-Erceg**

Croatia

**Feng-Yan Bai**

China

**Ilker Balkan**

Turkey

**Giuliana Banche**

Italy

**Tatiana Baranovich**

United States of America

**Ayse Batirel**

Turkey

**Lilly Bebora**

Kenya

**Gabor Bekesi**

Hungary

**Douglas Biedenbach**

United States of America

**Tannaz Birdi**

India

**Hurrem Bodur**

Turkey

**Francesca Bonvicini**

Italy

**Brandon Bookstaver**

United States of America

**Gülay Börekçi**

Turkey

**Laid Boukraa**

Algeria

**Bülent Bozdogan**

Turkey

**Irene Burckhardt**

Germany

**Elisa Burdino**

Italy

**Alfonso Carrillo-Muñoz**

Spain

**Sergio Casella**

Italy

**Elio Castagnola**

Italy

**Angeles Castro**

Spain

**Pauline Cavanagh**

Norway

**Aygul Celik**

Turkey

**Angkana Chaiprasert**

Thailand

**Arindam Chakraborty**

India

**Ben Chan**

Hong Kong

**Kai-chih Chang**

Taiwan

**Sheng Chen**

Hong Kong

**YW Chu**

Hong Kong

**Chun-Hung Chu**

Hong Kong

**Hueywen Chuang**

Taiwan

**Nuannoi Chudapongse**

Thailand

**Ihsan Hakki Ciftci**

Turkey

**Sylvie Dargere**

France

**Satadal Das**

India

**Dalia Mohamed Mohamed Dawoud**

United Kingdom

**Laura De Martino**

Italy

**Tuna Demirdal**

Turkey

**Lalitagauri Deshpande**

United States of America

**Maria Cecilia Dignani**

Argentina

**Jean-Denis Docquier**

Italy

**Arathy DS Nair**

United States of America

**Devrim Dundar**

Turkey

**Marthie Ehlers**

South Africa

**Nazif Elaldi**

Turkey

**Mohamed Elasri**

United States of America

**Katherine Ellingson**

United States of America

**Onder Ergonul**

Turkey

**Saban Esen**

Turkey

**Kiatichai Faksri**

Thailand

**Weihuan Fang**

China

**Rita Finley**

Canada

**Samantha Flores-Treviño**

Mexico

**Kazuyo Fujita**

Japan

**Ke-ichi Fujita**

Japan

**Neeta Gade**

India

**Abhijit Ganguli**

India

**Yvette Gbelska**

Slovakia

**Jianning Ge**

United States of America

**Serap Gencer**

Turkey

**K. Aaron Geno**

United States of America

**Evangelos Giamarellos-Bourboulis**

Greece

**Veronica Godoy**

United States of America

**Harumi Gomi**

Japan

**Maria Aparecida Grossi**

Brazil

**Ertugrul Guclu**

Turkey

**Maria Guembe**

Spain

**Iram Gull**

Pakistan

**Murat Gunaydin**

Turkey

**Paban Gürcan**

Turkey

**Ana María Guzmán Durán**

Chile

**Rand Hafidh**

Iraq

**Christine Hansen**

Denmark

**Sonja Hansen**

Germany

**Volkan Hazar**

Turkey

**Helmut Heinle**

Germany

**Janet Hindler**

United States of America

**Yoichi Hiraki**

Japan

**Murat Hokelek**

Turkey

**Salih Hosoglu**

Turkey

**Enzo Ierardi**

Italy

**George Jacoby**

United States of America

**Seok Hoon Jeong**

South Korea

**Christoph Jochum**

Germany

**Tineke Jones**

Canada

**Thomas Jové**

France

**Haider Kadhim**

Iraq

**Adel Kadri**

Tunisia

**Ahmet Kalkan**

Turkey

**Tatsuo Kanda**

United States of America

**Jia-Horng Kao**

Taiwan

**Oguz Karabay**

Turkey

**Stylianos Karatapanis**

Greece

**Samuel Kariuki**

Kenya

**Shunmugiah Karutha Pandian**

India

**Rejin Kebudi**

Turkey

**Kristina Keitel**

United States of America

**Abdul Khan**

South Korea

**Selcuk Kilic**

Turkey

**Jong Bae Kim**

South Korea

**Ioannis Kioumis**

Greece

**Petar Knezevic**

Serbia

**Esra Kocoglu**

Turkey

**Tee Kok Keng**

Malaysia

**Iftihar Koksal**

Turkey

**Mehmet Koroglu**

Turkey

**Sibel Kucukoglu**

Turkey

**Nishanth Kumar**

India

**Gabriel Kuty Everett**

United States of America

**Botond Lakatos**

Hungary

**Adebayo Lamikanra**

Nigeria

**Felix Lange**

Germany

**Janos Lantos**

Hungary

**Chia-Long Lee**

Taiwan

**Gianluigi Li Bassi**

Italy

**Adamantia Liapikou**

Greece

**Ming-Feng Lin**

Taiwan

**Yi-Tsung Lin**

Taiwan

**Ulrike Lindequist**

Germany

**Sonia Lopez Cuenca**

Spain

**Alexander Lukatkin**

Russian Federation

**Enis Macit**

Turkey

**Jane Maiamichael**

Saudi Arabia

**Piotr Majewski**

Poland

**Caterina Mammina**

Italy

**Dror Marchaim**

Israel

**Kalisvar Marimuthu**

Singapore

**Jose Martinez**

Spain

**Yasufumi Matsumura**

Japan

**Ahmet Mavi**

Turkey

**Sante Mazzacane**

Italy

**Matthew McCusker**

Ireland

**Meliha Meric Koc**

Turkey

**Tor Monsen**

Sweden

**Azucena Mora**

Spain

**Paula Morais**

Portugal

**Stephen Mshana**

Tanzania

**Diriba Muleta**

Ethiopia

**Hossein Nahrevanian**

Iran

**Kazuhiko Nakano**

Japan

**Rahul Nandre**

United States of America

**Mbulelo Ncango**

South Africa

**Penny Neave**

United Kingdom

**Billy Ngasala**

Sweden

**Somashekhar Nimbalkar**

India

**Yoshimi Niwano**

Japan

**Georges Offenstadt**

France

**Aziz Ogutlu**

Turkey

**Olusola Ojurongbe**

Nigeria

**Maria Leide Oliveira**

Brazil

**Pinar Ongürü**

Tuvalu

**Ronald Ordinola Zapata**

Brazil

**Maria Cristina Ossiprandi**

Italy

**Resar Ozaras**

Turkey

**Burcin Ozer**

Turkey

**Recep Ozturk**

Turkey

**Rabindra Padhy**

India

**Nora Lia Padola**

Argentina

**Bibhuti Bushan Pal**

India

**Joseph Papaparaskevas**

Greece

**Dane Parker**

United States of America

**Maria Pieters**

United States of America

**Antonio Pignatari**

Brazil

**Tatiana Pinto**

Brazil

**João Pires**

Portugal

**Garyphallia Poulakou**

Greece

**Bruno Pozzetto**

France

**Richard Pushkin**

United States of America

**Laura Puzniak**

United States of America

**Barbara Rasco**

United States of America

**Iraj Rasooli**

Iran

**Sara Reese**

United States of America

**Faiza Rezwan**

Saudi Arabia

**Vanessa Ribeiro**

Brazil

**Nadia Roan**

United States of America

**Ashley Robinson**

United States of America

**Heinz Rode**

South Africa

**Manuel Rodriguez-Iglesias**

Spain

**Giampiero Rossi Fedele**

United Kingdom

**Eduardo Ruiz-Bustos**

Mexico

**Yolanda Sáenz**

Spain

**Vikram Saini**

United States of America

**Adekunle Sanyaolu**

Anguilla

**Andreas Sauerbrei**

Germany

**Frieder Schaumburg**

Germany

**Domenico Schillaci**

Italy

**Salome Seiffert**

Switzerland

**Saikat Sen**

India

**Chaminda Jayampath Seneviratne**

Singapore

**Toshiro Shirakawa**

Japan

**Hanna Sidjabat**

Australia

**Sónia Silva**

Portugal

**Balbir Singh**

Malaysia

**Nidhi Singla**

India

**Sinead Smith**

Ireland

**Gloria Soberon-Chavez**

Mexico

**Marcos Soto-Hernandez**

Mexico

**Vidya Sundareshan**

United States of America

**Suheyla Surucuoglu**

Turkey

**Morovat Taherikalani**

Iran

**Hern Tze Tan**

Malaysia

**Vishnu Tandon**

India

**Zhan Hong Tang**

China

**Abdul Malik Tareen**

Pakistan

**Johan Tham**

Sweden

**Enty Tjoa**

Indonesia

**Marcello Trevisani**

Italy

**Yumiko Tsukamoto**

Japan

**Tafese Beyene Tufa**

Ethiopia

**Nazan Tuna**

Turkey

**Maurizio Turiel**

Italy

**Edet Udo**

Kuwait

**Glen Ulett**

Australia

**Cemal Ustun**

Turkey

**David van Duin**

United States of America

**Leo van Griensven**

Netherlands

**Christina MJE Vandenbroucke-Grauls**

Netherlands

**Alkiviadis Vatopoulos**

Greece

**Vania Vicente**

Brazil

**Nicolas Vignier**

France

**Rafael Vignoli**

Uruguay

**Sofia Vourli**

Greece

**Lishi Wang**

United States of America

**Micael Widerstrom**

Sweden

**Michael Wink**

Germany

**Robert D. Wojtyczka**

Poland

**Qiaobin Xiao**

United States of America

**Gangming Xu**

China

**Chuan Shan Xu**

Hong Kong

**Meng Xun**

China

**Pablo Yagupsky**

Israel

**Kuo-ming Yeh**

Taiwan

**Gürdal Yilmaz**

Turkey

**Mesut Yilmaz**

Turkey

**Heather Young**

United States of America

**Yunsong Yu**

China

**Roberto Zenteno-Cuevas**

Mexico

**Kui Zhang**

China

**Xihong Zhao**

China

**Heng Zheng**

China

